# Evidence of High Fluorine Ion Conductivity in SrF_2_-Rich SrF_2_–TiO_2_-Based Compounds

**DOI:** 10.1021/acsomega.5c02253

**Published:** 2025-04-28

**Authors:** Yatir Sadia, Gwilherm Kerherve, Stephen J. Skinner

**Affiliations:** † Material Engineering, Ben Gurion University of the Negev, P.O. Box 653, Beer-Sheva 8410501, Israel; ‡ Faculty of Engineering Materials Engineering, Bar Ilan University, Ramat Gan 5290002, Israel; § Department of Materials, Imperial College of London, South Kensington Campus, London SW7 2AZ, U.K.

## Abstract

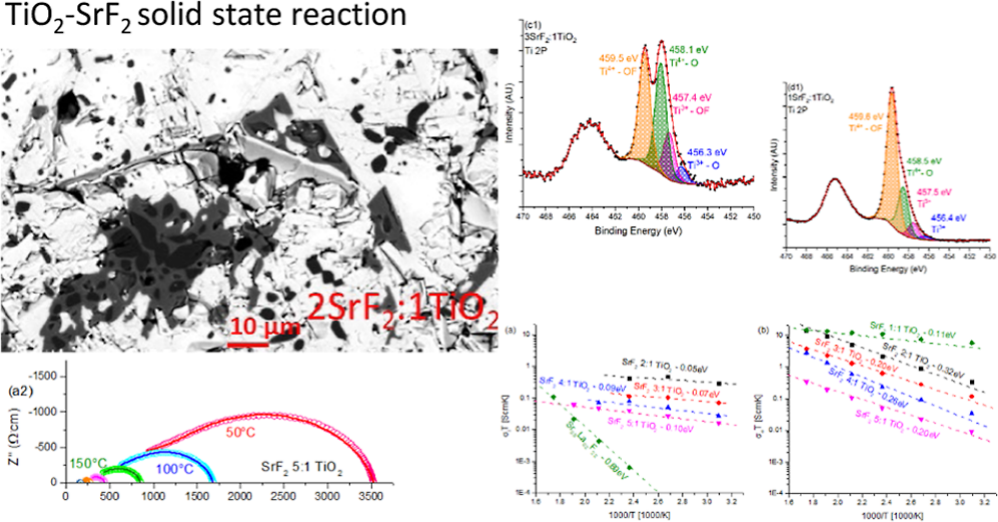

Materials based on Sr–Ti–O have shown remarkable properties and have been used in a wide diversity of applications. However, very little investigation has gone into the Sr–Ti–O–F system, mainly due to the very high stability of its constituents such as SrF_2_ and TiO_2_. In this work, solid-state reactions under reducing atmospheres on SrF_2_ and TiO_2_ showed highly interesting properties for the system, including high mixed electronic and ionic conductivity. XPS data further illuminate the results with the Ti 2p peaks shifting to higher binding energy due to fluorine interaction, possibly hinting at the formation of a TiO_*x*_F_*y*_ phase. Testing using both ion blocking and electron blocking layers allowed the distinction between the ionic and electronic conductivity of the materials, showing very high ionic conductivity compared to most SrF_2_-based compounds. Ionic conductivities of up to 1 mS cm^–1^ for 2SrF_2_:TiO_2_ samples near room temperature were obtained. The ionic transport numbers were found to be 20–60% over the 50–300 °C temperature range. The apparent activation energy for ionic conduction was surprisingly low with *E*_a_ (ionic) = 0.05–0.10 eV for the different samples, whereas for electronic conductivity, *E*_a_ (electron) = 0.11–0.32 eV.

## Introduction

Oxyfluoride materials are some of the most interesting materials for various applications. Oxyfluorides have been identified as interesting for battery applications for both fluoride-ion batteries^1−3^ and lithium-ion batteries.^4−6^ Oxyfluorides have been shown to conduct both oxygen^7,8^ and fluorine^9,10^ and have been identified as both superconductors^11,12^ and materials for transparent electronics.^13,14^ Some of the most interesting oxyfluorides come from systems where the composition is based on a perovskite such that AB(O,F)_3_ is the representation of the material.^2,15,16^ As such, SrTi(O,F)_3_ might be enticing for research. Very little research has been done on SrTiO_*x*_F_*y*_ based materials, with most of it being achieved through fluorinating Sr_2_TiO_4_^17,19^ and Sr_3_Ti_2_O_7_.^18^ This is done mainly using chemical fluorination using either NH_4_F^17^ or PVDF.^18^ However, high-temperature synthesis from stable compounds such as TiO_2_ and SrF_2_ by reaction of the materials has not been thoroughly investigated.

SrF_2_ is an insulating ceramic with a very wide band gap of 7.12 eV^20^ and negligible ionic conductivity below 1000 °C.^21^ Most applications for SrF_2_ have been based on optical uses for UV and VUV spectroscopy.^22^ With the correct doping, SrF_2_ can be a good fluorine ion conductor. Doping SrF_2_ with Ce,^23^ Y and Na,^24^ and Yb^25^ achieved a maximum conductivity of 4.4 × 10^–8^ S·cm^–1^ at 227 °C with 10% Na, 0.4% Y, and 20.8% Yb and approximately 1.1 × 10^–6^ S·cm^–1^ for 23% Ce substitution at 127 °C.^23−25^ The best dopant for SrF_2_ seems to be La, with most conductivities reported as being around 8 × 10^–2^ S·cm^–1^ at 300 °C with 20% La^26^ which was used as a reference material in this study. Conduction in SrF_2_-based materials is almost purely ionic, with the electronic conductivity being 2–3 orders of magnitude lower. In SrF_2_, the activation energy for conduction through fluorine vacancies is 0.4–0.7 eV,^26^ while conduction through fluorine interstitials is closer to 0.75–0.95 eV.^26^ Fluorine conductors can serve several applications, such as fluoride ion batteries, separation membranes for fluorine extraction, and fluorine detectors. While doped SrF_2_ is a decent fluoride ion conductor, many fluoride ion conductors are much better conductors. The most studied are tysonite,^27−29^ fluorite,^30−32^ perovskite,^33−35^ and amorphous glasses;^36−38^ the most conductive samples are mainly based on MSnF_4_-type materials.^39−43^ By far, the best conductivity is shown by the PbSnF_4_-based material in the β-structure phase.^40−43^ The highest conductivities near a room temperature of 300 K are about 0.1 S·cm,^40−43^ whereas the best perovskites such as CsPb_0.9_K_0.1_F_2.9_ reach about 0.01 S·cm at 300 K.^33,34^

TiO_2−δ_ is an insulating ceramic with a band gap of 1.86 eV^44^ and negligible electronic and ionic conductivity when δ = 0.^45^ However, when produced in a reducing environment, TiO_2−δ_ can show a wide range of electronic conductivities from slightly to highly conductive.^45−49^ When reduced, oxygen vacancies in TiO_2−δ_ are compensated by excess electrons at first, leading to Ti^3+^ sites. Further increases in reduction introduce Ti^3+^ interstitials as well. In both regimes, however, the conduction is attributed to electrons, which are the major species. Oxygen ion conduction is very low for TiO_2_-based materials, reaching only 10^–4^ S·cm^–1^ at 892 °C.^45^ This figure is actually constant in different oxygen concentrations, as the compensation mechanisms in TiO_2_ are based on electron and hole compensation, leading to electronic conductivity.

TiO_*x*_F_*y*_-type materials have been previously investigated, and they show high potential as photocatalysts,^50−53^ as lithium-ion conductors for intercalation as anodes in lithium batteries,^54−57^ and as electrochemical and super capacitors.^58,59^ TiOF_2_ is the stable phase discussed in all papers related to titanium oxyfluorides. TiOF_2_ is hexagonal with a hexagonal to cubic transition near 50–60 °C.^60^ It is conductive for both electrons and lithium ions and is a route to obtain different morphologies of anatase.^61,62^

SrTiO_3_ is one of the semiconductors with the most diverse applications, finding uses in thermoelectric materials,^63−65^ fuel cells,^66−68^ biodiesel production,^69−71^ and more. SrTiO_3_-based materials, such as the layered perovskites of general composition (Sr_*n*+1_Ti_*n*_O_3*n*+1_), show a semiconducting behavior with a band gap of 3.25 eV for SrTiO_3_, 3.5 eV for Sr_2_TiO_4_, and 3.4 eV for Sr_3_Ti_2_O_7_.^72^ From the diverse properties of SrF_2_, TiO_2_, TiOF_2_, and SrTiO_3_, it is expected that any “SrTiO_*x*_F_*y*_”-type material should have interesting electrical properties and therefore direct reaction of SrF_2_ and TiO_2_ using a solid-state reaction should yield new materials with interesting functionality.

## Methodology

### Sample Preparation

SrF_2_ (99.9%) and TiO_2_ (99.8% TiO_2_) powders purchased from Alfa Aesar were mixed in the ratios of 5:1, 4:1, 3:1, 2:1, and 1:1, respectively. Either 5 or 10 g of the mixtures were pressed in a 30 mm diameter graphite die using BN as a separator to produce 1.5 or 3 mm thick samples after grinding accordingly. The samples were heated at 13 °C/min to 800 °C and at 7.5 °C/min up to 1100 °C. The pressure was increased to a maximum of 28 MPa; at 1100 °C, the pressure was then slowly decreased to 2.8 MPa during 5 h of maintaining a temperature of 1100 °C. At this stage, the sample was cooled slowly to room temperature at 10 °C/min without pressure. The entire process was done in an argon UHP atmosphere with a custom-made graphite hot press from FCT GmbH (FCT, Germany). The samples were then ground to remove any traces of BN and graphite, and neither of these species was detected using energy-dispersive X-ray spectroscopy (EDS) or X-ray diffraction (XRD).

La_0.2_Sr_0.8_F_2.2_ samples were produced by mixing SrF_2_ powder with LaF_3_ powder (99.9%) from Alfa Aesar in the relevant ratio and pressing them in the hot-press using the same conditions as the SrF_2_–TiO_2_ samples, while the layered samples were prepared by putting the mixed SrF_2_–LaF_3_ powders into the graphite die, then slightly pressing, then adding the SrF_2_–TiO_2_ samples and slightly pressing, and finally adding another layer of the La_0.2_Sr_0.8_F_2.2_ and running the same process as mentioned above.

### Characterization

XRD data were collected using a Panalytical Empyrean diffractometer with an X’Celerator detector with a Cu Kα source (λ = 1.5405 Å) operating at 40 kV and 30 mA, with analysis performed using the GSAS-II software package.^73^ Scanning electron microscopy (SEM) was done with a JEOL-5600 operating at 15 keV using a backscattered electron detector (JEOL, Japan). Energy-dispersive X-ray spectroscopy was obtained using a Noran EDX-System (Thermo Fisher Scientific, USA).

X-ray photoelectron spectroscopy (XPS) was performed using a high-throughput X-ray photoelectron spectrometer (K-Alpha+, Thermo Fisher Scientific, US) with a monochromated Al Kα radiation source (*h*ν = 1486.6 eV) operating at a 2 × 10^–9^ mbar base pressure. The X-ray source used a 6 mA emission current and a 12 kV anode bias, giving an X-ray spot size of up to 400 μm^2^. Survey and core-level spectra were obtained with pass energies of 200 and 35 eV, respectively. Spectra were processed by subtraction of a Shirley-type background, and peaks were fitted using a Gaussian–Lorentzian line shape.

### Electrochemical Impedance Spectroscopy (EIS)

EIS was performed by sputtering gold electrodes on both sides of 10 × 10 × 1 mm samples. Each sample was heated to 300 °C in 50 °C steps using a Carbolite tube furnace (Carbolite, UK) and a home-built rig, letting the samples equilibrate at each temperature for 1 h. The measurements were taken under the flow of Ar. The measurements were obtained over the frequency range of 1 MHz to 0.1 Hz using a Solartron 1260 Frequency Response Analyzer (Solartron, UK) with an AC amplitude of 100 mV. Pt wires and mesh on the rig were used for current collection. Data analysis was conducted using ZView4 software (Scribner, USA).

## Results

### Microstructure

**Figure 1:**
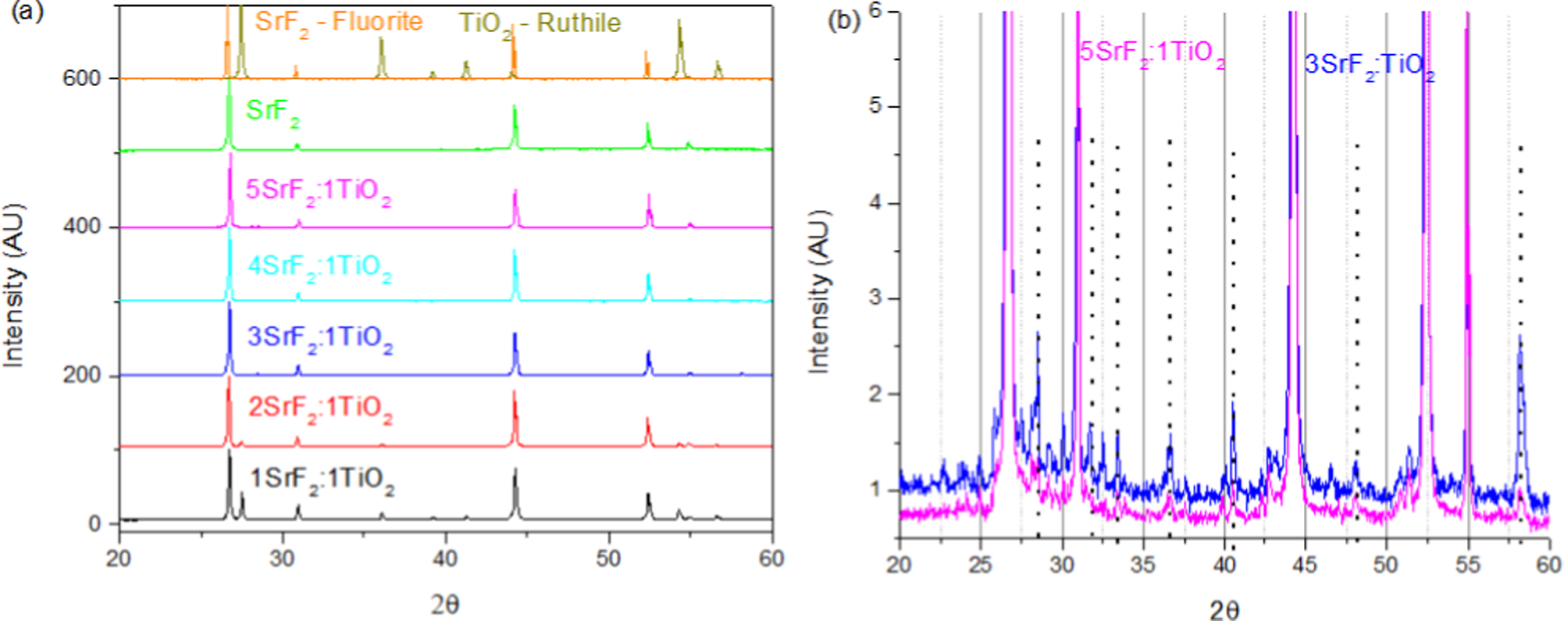
(a) XRD results for SrF_2_:TiO_2_ composites with the theoretical rutile and fluorite phases shown above (b), showing the small peaks of a secondary phase marked by dotted lines in 3SrF_2_:1TiO_2_ and 5SrF_2_:1TiO_2_; none of these peaks are above 3% intensity.

**Figure 2:**
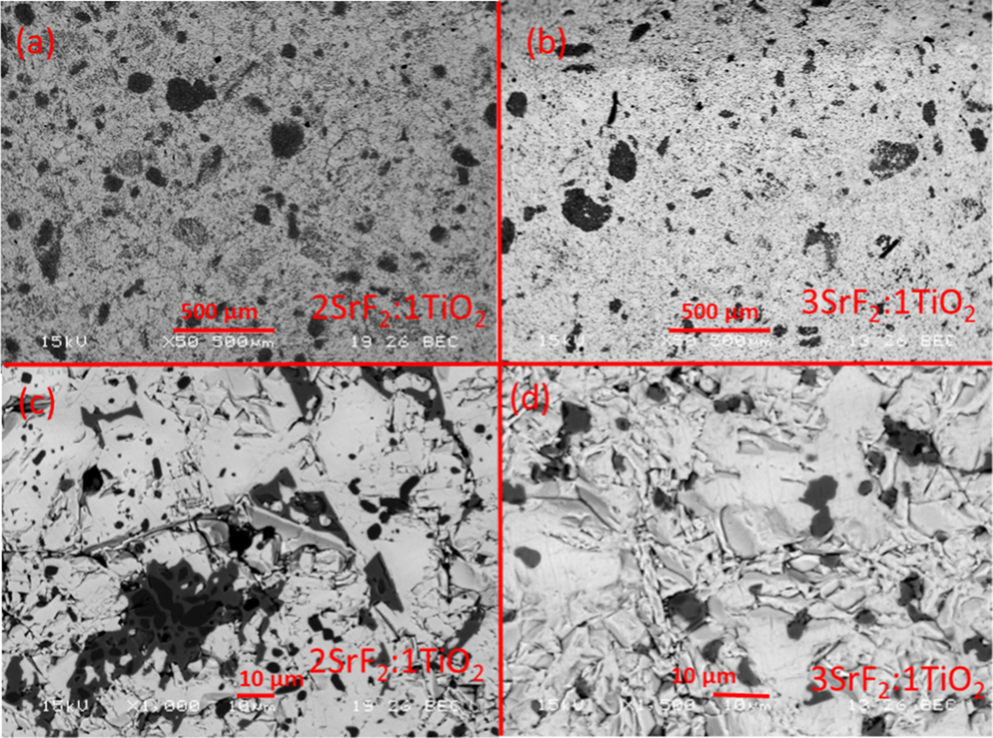
SEM micrographs obtained in backscattered electron mode. (a) and (b) showing 2SrF_2_:1TiO_2_ showing 3 phases, TiO_2_ in black embedded in an unknown gray phase and SrF_2_ in white. (c) and (d) showing 3SrF_2_:1TiO_2_ showing 3 phases, an unknown gray phase and SrF_2_ in white. The unknown gray phase is about 10–15% SrF_2_ and 85–90% TiO_2_ based on EDS analysis.

All samples showed a density of 4.1–4.2 g/cm^3^, where 4.23 and 4.24 g/cm^3^ are the theoretical densities of TiO_2_ and SrF_2_, respectively. All samples showed up to 3 distinct phases: SrF_2_, TiO_2−δ_, and a small amount of an additional phase which contains mainly TiO_*x*_, based on EDS analysis, but could not be identified by XRD as any known TiO_2_ or Ti_*x*_O_*y*_ phase. Predicted XRD peaks of the SrF_2_ and TiO_2_ phases are shown in Figure 1a. Examining a selected 2θ range, shown in Figure 1b, highlights small peaks at 28.51, 36.49, 40.55, and 58.12° 2θ shown here for 5-Sr:1-Ti and 3-Sr:1-Ti. However, no known phase is associated with these peaks, and their low intensity in the XRD pattern of under 3% for the highest intensity peak confirms this as a minor impurity phase. As shown in Figure 2d, SEM confirms that this unidentified phase is present in only small quantities with little to no connectivity, which is relevant for percolation. SEM backscattered micrographs are shown in Figure 2a–d for (a,c) 2-Sr:1-Ti and (b,d) 3-Sr:1-Ti. The white phase in Figure 2c is SrF_2_, and the black phase is TiO_2_, with the gray phase representing the unknown phase. In Figure 2d, only the white (SrF_2_) and gray phases are shown as expected based on the XRD results. In Table 1, the ratio of the two main phases and the lattice parameters of the phases are shown. Little to no difference in the lattice parameters is seen. In places where the TiO_2_ results were within the measurement error, they are marked in red, showing that they are present in the same amount as in the SrF_2_-only sample, which contained no TiO_2_. The density of all samples was found to be 4.2 g/cm^3^ as measured by the Archimedes method, showing that samples had achieved a density of at least 98% of the theoretical value.

**Table 1:**
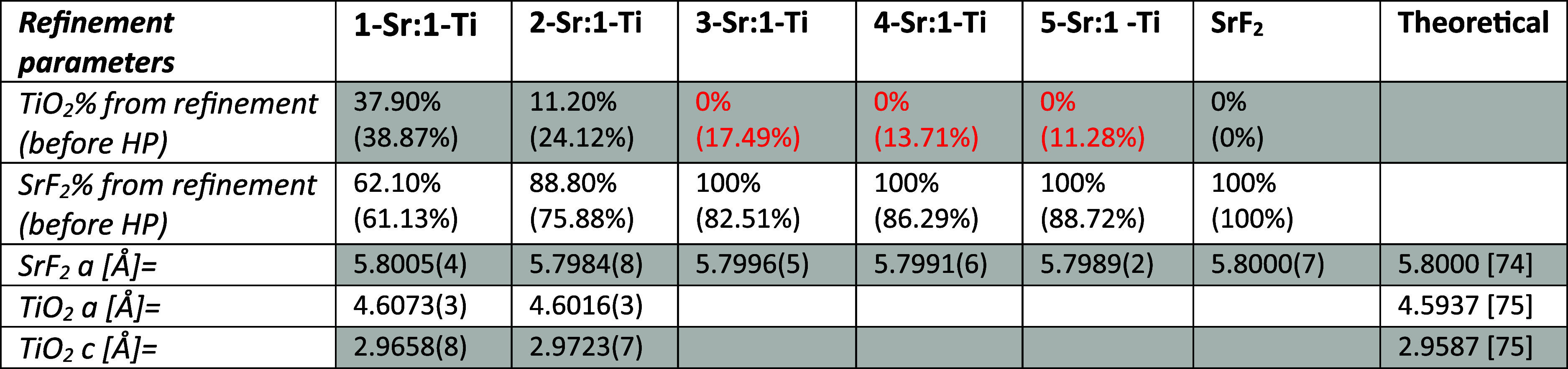
Fraction of TiO_2_ and SrF_2_ Obtained by Rietveld Refinement of XRD Data, with the Values for TiO_2_ and SrF_2_ Powders Put into the Sample before Hot Pressing by Weight Marked in Parentheses to Give a Reference for the Change^a^

a Samples showing no TiO_2_ after hot pressing (HP) are marked in red.

### X-ray Photoelectron Spectroscopy (XPS)

**Figure 3:**
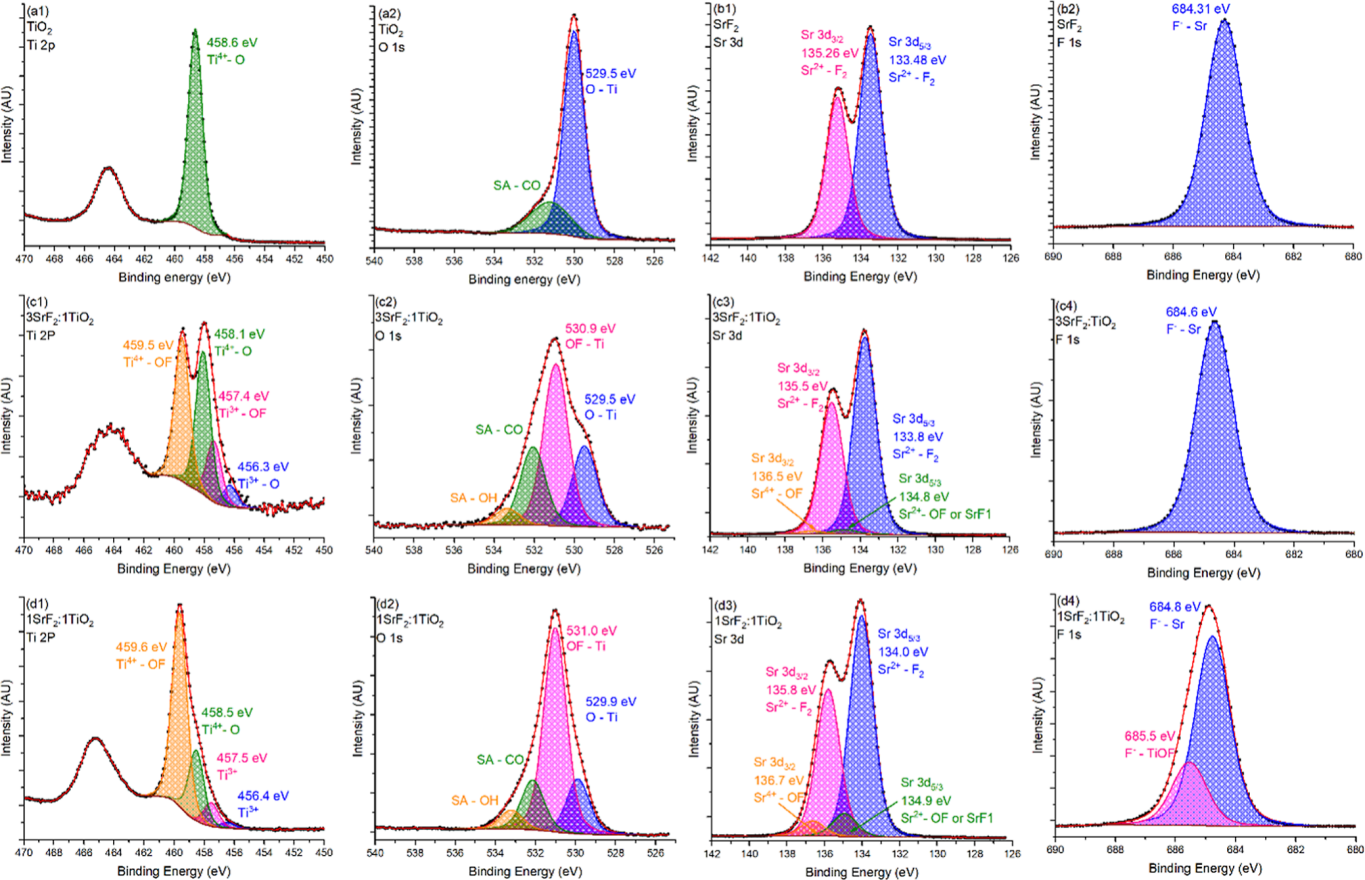
XPS of the (a) TiO_2_ reference sample, (b) SrF_2_, (c) 3SrF_2_:1TiO_2_, and (d) 1SrF_2_:1TiO_2_ with 1 Ti 2p, 2 O 1s, 3 Sr 3d, and 4 F 1s; here black is the original data, red is the fitting, and the rest of the colors are different peaks marked in the figure.

XPS data were obtained for 1SrF_2_:1TiO_2_ and 3SrF_2_:1TiO_2_ initial ratios, along with the reference SrF_2_ and TiO_2_ samples produced in the same manner. Figure 3 shows the XPS results of the samples for (1) Ti 2p, (2) Sr 3d, (3) O 1s, and (4) F 1s. All samples have been corrected for the C–C C 1s feature at 284.8 eV. There are a number of initial key observations from the XPS analysis.

The highest energy Ti 2p feature is at 459.5 eV for the 3SrF_2_:1TiO_2_ and 1SrF_2_:1TiO_2_ samples. This is at a higher binding energy than expected from the reference TiO_2_ sample with no SrF_2_, which is at 458.6 eV. This agrees with literature data^60,76,77^ for Ti^4+^ in some configuration of TiO_*x*_F_*y*_, identified as spectral features at 458.9–459.1 eV. This is understandable as Ti^4+^ from TiO_2_ is observed at 458.6 eV and at 461.1 eV for TiF_4_ as defined by Klimov et al.^78^ Surprisingly, the TiO_2_ sample shows only a negligible amount of Ti^3+^, whereas the SrF_2_:TiO_2_ samples show Ti^3+^ in both the oxide state (Ti_2_O_3_) and the oxyfluoride (TiO_*x*_F_*y*_) state. This suggests that a higher number of vacancies were created in the Ti-rich phase. It also points to the Ti-rich phase in SEM as some unknown form of TiO_*x*_F_*y*_ that does not fit a known XRD pattern. It is noteworthy that the ratio of oxide to oxyfluoride seems to be higher in the 1:1 ratio sample, whereas in the 3:1 sample, the ratio of oxide to oxyfluoride is about 1. The O 1s core level spectra show a shift toward higher binding energies going from 529.5 eV to about 531 eV, in agreement with previously reported oxyfluoride behavior.^76^ Higher energy peaks associated with CO and OH were ignored as surface effects.

As for the Sr 3d peaks, SrF_2_ showed a doublet at 133.5 and 135.3 eV, whereas the samples that contained TiO_2_ as well showed another doublet at around 134.9 and 136.6 eV. A similar increase of the binding energy due to the presence of TiO_2_ has been shown by Subash et al.,^79^ and in epitaxial SrF_2_,^80^ Singh et al. have also attributed a peak at 136.5 eV to the SrF_2_ reaction with oxygen in thin films, and fluorine doping in SrTiO_3_ due to a reaction with HF has also been shown to increase the binding energy to 136.2 eV.^81^ As for the fluorine peaks, only the 1SrF_2_:1TiO_2_ sample shows a major peak movement; this makes sense as this sample shows the most pronounced TiO_*x*_F_*y*_ in the Ti 2p spectrum and the most satellite peaks at 134.9/136.6 eV in the Sr 3d spectra.

From XPS, it is clear that (1) there is some formation of a TiO_*x*_F_*y*_ type of phase which is more pronounced with greater TiO_2_ content in the system. (2) The SrF_2_ phase is shifted to higher energies for both F and Sr due to either loss of fluorine or exchange of fluorine with oxygen.

### Electronic and Ionic Conductivities

Samples were coated with sputtered gold on both sides to provide F^–^ blocking layers.^82^ The samples were measured at temperatures of up to 300 °C in steps of 50 °C.

**Figure 4:**
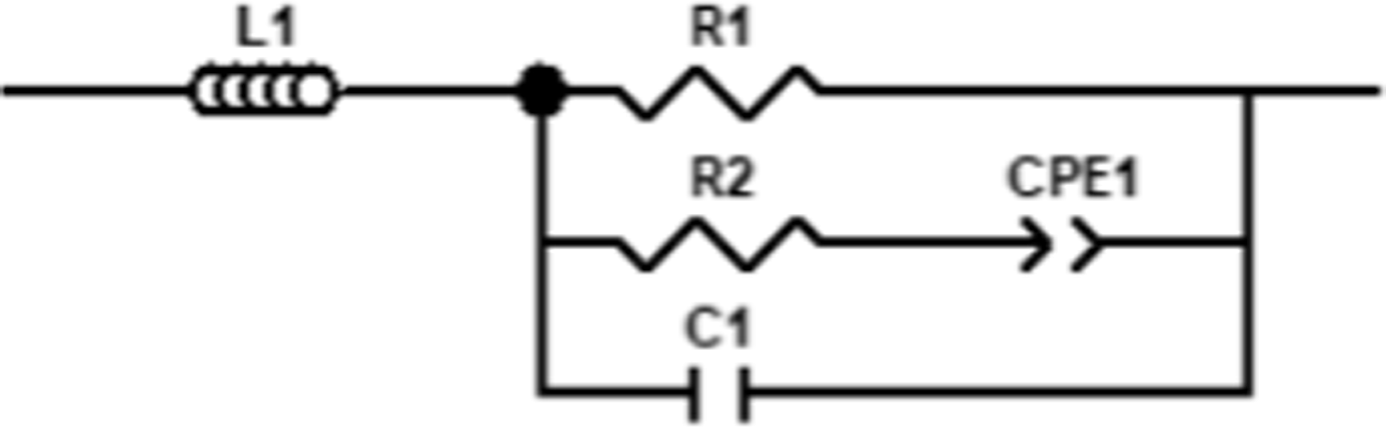
Short ion-conducting circuit with ion-blocking layers.

The simplest circuit that fit most of the data was a shortened circuit, as seen in Figure 4. This circuit is based on the analysis by Huggins^83^ with the electronic conductor marked by R_1_ and an ionic conductor with blocking electrodes marked by R_2_ and CPE_1,_ representing the resistance of the ionic conductor and the capacitance due to the blocking electrodes. An induction element was added to account for the wiring of the rig as suggested by Joncher.^84^

**Figure 5:**
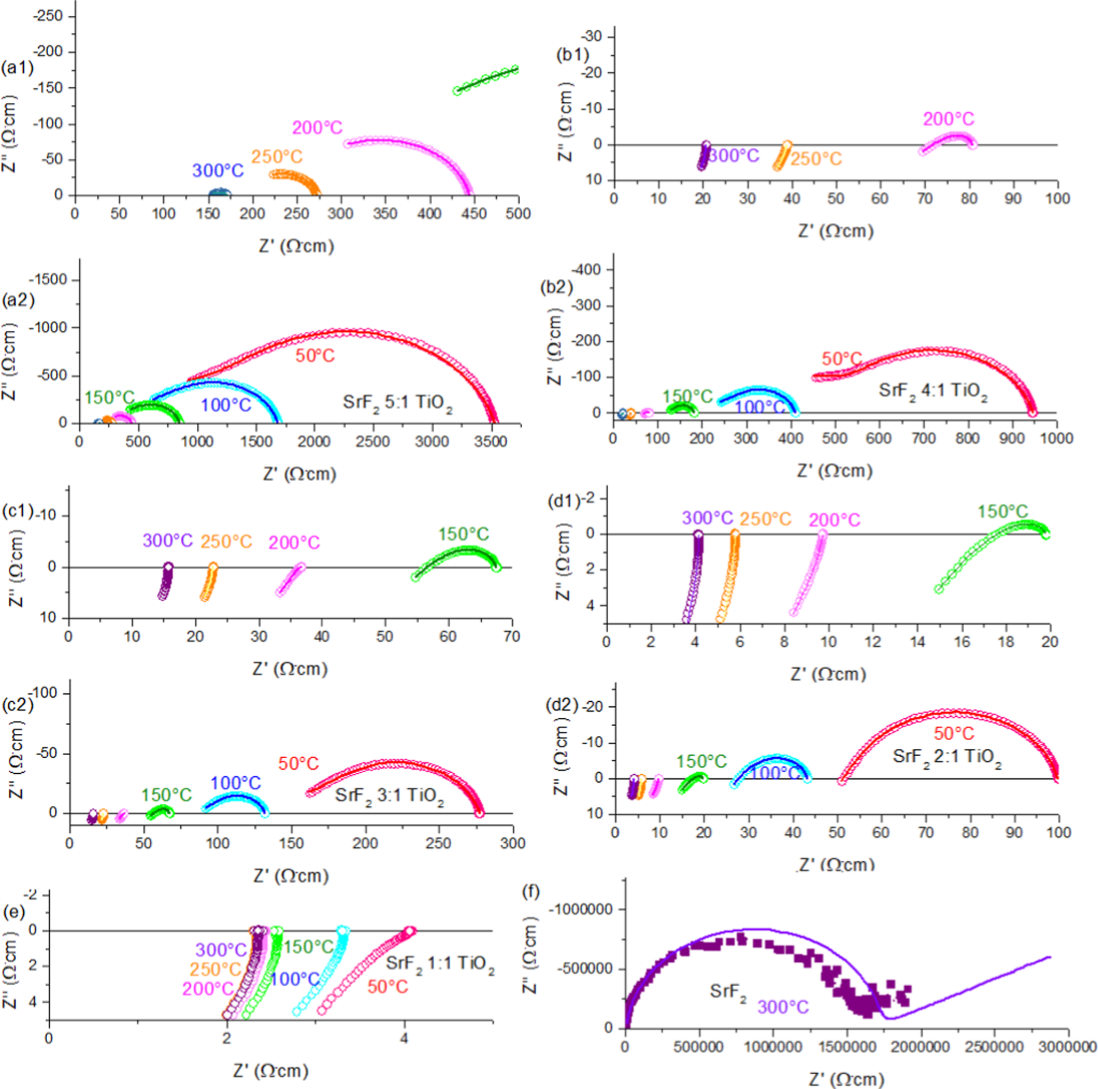
EIS spectra of all samples shown as Nyquist plots (a1–2) SrF_2_ 5:1 TiO_2_ and (b1–2) SrF_2_ 4:1 TiO_2_, (c1–2) SrF_2_ 3:1 TiO_2_ and (d1–2) SrF_2_ 2:1 TiO_2_, (e) SrF_2_ 1:1 TiO_2_, and (f) SrF_2_ only. Color coded so that the results are in dashes with light red (50°), blue (100 °C), green (150 °C), magenta (200 °C), orange (250 °C), and purple (300 °C). The model results are in dotted dark red (50°), blue (100 °C), green (150 °C), magenta (200 °C), orange (250 °C), and purple (300 °C).

In Figure 5, the Nyquist plots of the EIS results for all samples are shown. These data are color-coded so that the results are in dashed lines with light red (50°), blue (100 °C), green (150 °C), magenta (200 °C), orange (250 °C), and purple (300 °C). The model results are in dotted dark red (50°), blue (100 °C), green (150 °C), magenta (200 °C), orange (250 °C), and purple (300 °C) markers. It can be seen that conductivity continued increasing with additional TiO_2_ content. In addition, other than the 5:1 sample, all samples reach a temperature where the electronic conductivity far exceeds the ionic conductivity, resulting in complete electronic conductivity. The 4:1 sample turns mostly electronic between 200 and 250 °C, whereas for 3:1 and 2:1 that happens between 150 and 200 °C. With the 1:1 sample at all temperatures, the sample is mainly electronically conductive. Figure 5f shows the EIS of the SrF_2_ sample to show that the ionic and electronic conductivity are not a simple result of combining electronically conductive TiO_2−δ_ and ionically conductive SrF_2_ as SrF_2_ is barely conductive in this temperature range. The results are very “shaky”, and the model is not a perfect fit like in the other circuits. This is due to the fact that pure SrF_2_ is a very bad F-conductor even at 300 °C. This further proves that this is not the conduction source.

The short circuit is only relevant for transference numbers of above 10% ionic, meaning the ionic and electronic conductivities are within the same order of magnitude. In Table 2, the percentage of the ionic part of the transference number is shown. At any temperature where there were no results, the electronic conductivity was too high, and the ionic part was ignored in the analysis. The transference numbers are calculated to be between 64% and 10%.

**Table 2:** Transference Numbers of the Ionic Species (F^–^) vs the Overall Conductivity in All Samples

	transference number, τ_*i*_ %			
Temperature (°C)	2-Sr:1-Ti	3-Sr:1-Ti	4-Sr:1-Ti	5-Sr:1-Ti
50	46.1	37.1	44.2	63.7
100	33.8	26.6	35.7	55.0
150	14.9	14.8	24.8	44.3
200			10.5	30.7
250				24.3

**Figure 6:**
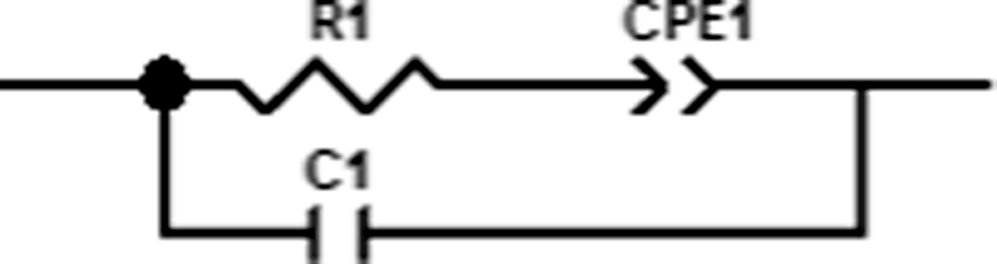
Blocking layer circuit.

To ensure that the ionic conductivity measured is not a different feature, electron-blocking layers of La_0.2_Sr_0.8_F_2.2_ were used on both sides of the sample. Since La_0.2_Sr_0.8_F_2.2_ blocks electrons and the gold layer blocks ions, the blocking layer analysis was performed using a regular non-shorted circuit seen in Figure 6, where R1 is the sum of the ionic resistance only of all 3 layers.

**Figure 7:**
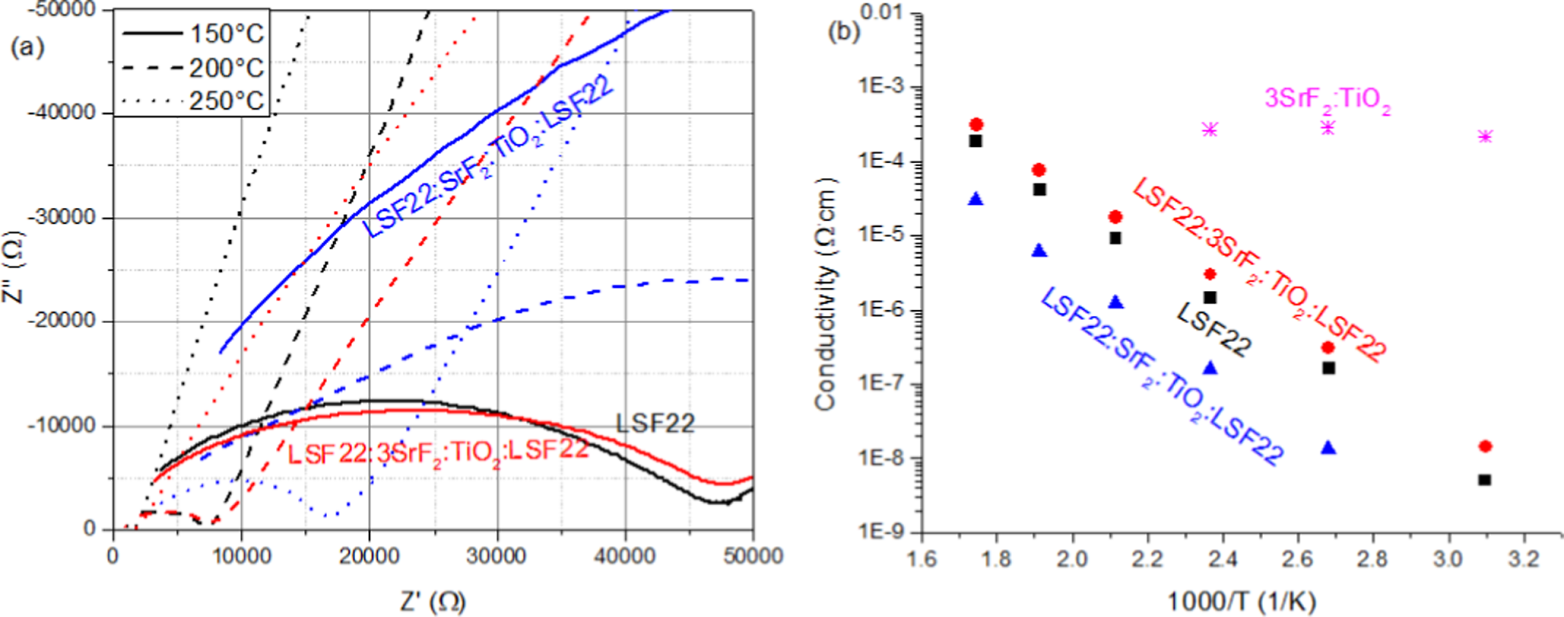
(a) EIS results from the samples with the blocking electrodes. La_0.2_Sr_0.8_F_2.2_ in black, layered La_0.2_Sr_0.8_F_2.2_: 3SrF_2_:TiO_2_:La_0.2_Sr_0.8_F_2.2_ in red, and La_0.2_Sr_0.8_F_2.2_: 3SrF_2_:TiO_2_:La_0.2_Sr_0.8_F_2.2_ in blue. Data recorded at 150 °C shown as solid lines, 200 °C in dashed and 250 °C in dotted lines. (b) Summary of the ionic conductivity results as a function of temperature from the EIS data along with the values derived from the 3SrF_2_:TiO_2_ sample added from previous results in magenta.

Figure 7a shows the EIS data, while Figure 7b presents the conductivity results of La_0.2_Sr_0.8_F_2.2_ (in black) and layered samples of 3SrF_2_:TiO_2_ in red and SrF_2_:TiO_2_ in blue. It is clear from both Figure 7a,b that 3SrF_2_:TiO_2_ is much more conductive than La_0.2_Sr_0.8_F_2.2_, showing the same conductivity even with the extra layer of 3SrF_2_:TiO_2_ in between the La_0.2_Sr_0.8_F_2.2_ layers; this agrees with the results from the EIS data presented in Figure 4c and shown in magenta in Figure 7b. However, SrF_2_:TiO_2_ does not conduct F^–^ as well as seen from Figure 7a,b, showing that there is a 1 order of magnitude lower conductivity in SrF_2_:TiO_2_ than in La_0.2_Sr_0.8_F_2.2_.

**Figure 8:**
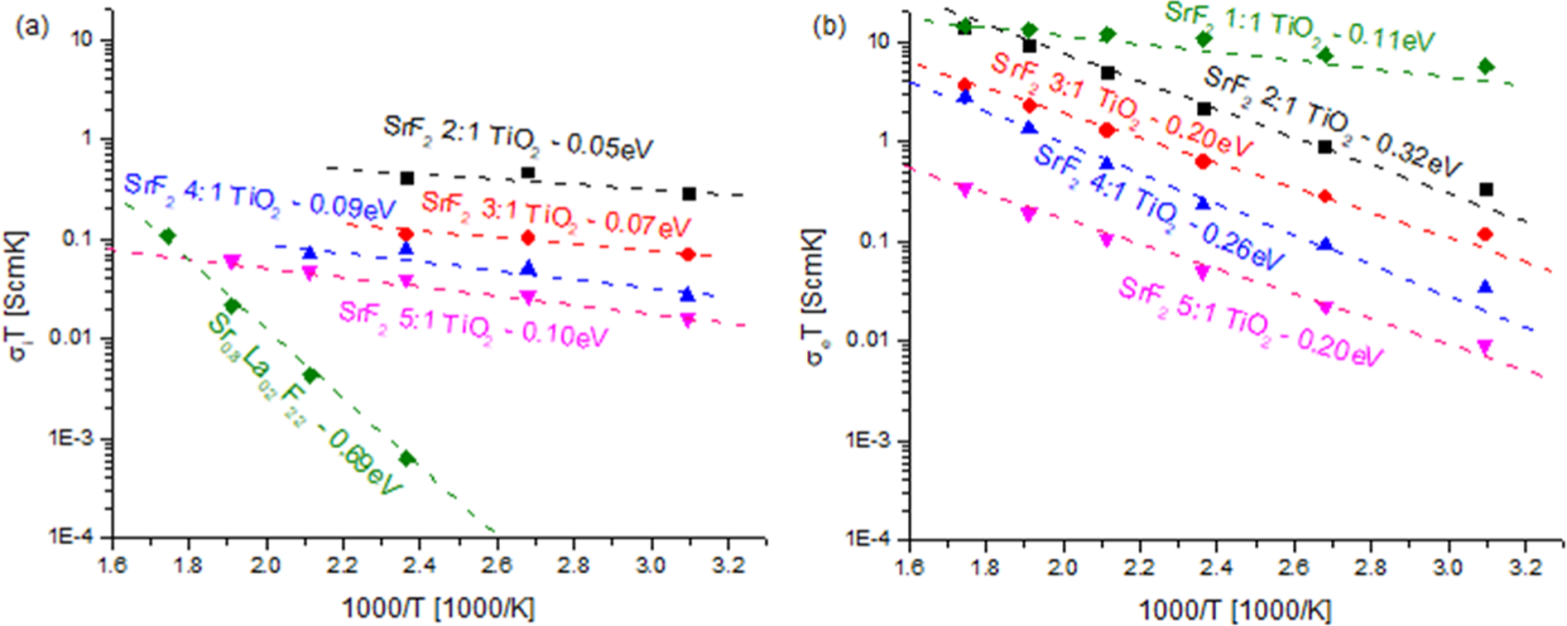
(a) σ*T*_ionic_ vs reciprocal temperature showing linear behavior of the activation energy for the SrF_2_:TiO_2_ series. (b) σ*T*_electronic_ vs reciprocal temperature showing linear activation energies for all samples.

In Figure 8, we can see the electronic and ionic conductivities for all samples calculated based on the circuit shown in Figure 4. All samples show linear behavior for log(σ*T*) vs 1/*T*, allowing an activation energy to be determined for all samples, which is shown in Figure 8b. In Figure 8a, the ionic conductivity is shown for all samples and temperatures at which the ionic conductivity was significant enough to separate from the electronic conductivity. In all samples, the activation energy was higher for the electronic conductivity than the ionic conductivity, preventing the measurement of ionic conductivity at high temperatures.

## Discussion

It can be seen that SrF_2_–TiO_2_-based materials show different properties than either TiO_2_ or SrF_2_. It seems that electronic conductivity arises from excess Ti^3+^ in the material from reduction of TiO_2_, while ionic conductivity arises from a combination of a SrF_2_ phase and a TiO_*x*_F_*y*_ phase. The electronic activation energy is of the order of 0.2 eV for the samples that contain no TiO_2−δ_, about 0.32 eV for samples that contain small amounts (11%) of TiO_2−δ_, and 0.1 eV for the sample that contains TiO_2−δ_ above 30% as is usually needed for percolation.^85,86^ This is an indication that the electronic conductivity is through the TiO_*x*_F_*y*_ phase at low TiO_2_ contents, whereas for high TiO_2−δ_ content, the conduction is through the TiO_2−δ_ phase. Another such explanation is that the conduction is increased at the interface between TiO_2−δ_ and SrF_2_ due to space charge effects of F^–^ accumulation at the TiO_2−δ_ vacancies created by the reduction of TiO_2−δ_. This had been discussed in depth by Li et al.^87^ This would explain why the highest conductivities are seen the closer we get to a 1:1 ratio of TiO_2−δ_ and SrF_2_.

The activation energy of the ionic conductivity of La_0.2_Sr_0.8_F_2.2_ was observed to be the same as the literature with within error at 0.69 eV.^88^ The activation energies for the SrF_2_:TiO_2_-based materials were very low and much lower than those previously reported for the SrF_2_-type materials. This is another indication that the conduction is mainly through a different phase, such as a TiO_*x*_F_*y*_ phase. With the growth of the TiO_2−δ_ phase, a reduction in the ionic conductivity begins, as seen by the samples with LSF electron blocking layers. The activation energies of 0.05–0.10 eV are closer to those found in β-PbSnF_4_ (0.2 eV)^39^ and KSn_2_F_5_ (0.24 eV) at high temperatures^89^ and 0.16 eV in the high-temperature phases in RbPbF_3_. Even activation energies below 0.1 eV are seen on K-doped β-PbSnF_4_^39^ and in CsPb_0.9_K_0.1_F_2.9_.^33,34^ Such an extremely low activation energy is interesting, especially for room-temperature applications such as batteries, which require constant resistance across a wide range of temperatures. It is noteworthy that while the TiO_2−δ_ phase might have some amorphous qualities as it is unseen by XRD up to the 2:1 ratio sample, it is not common that amorphous materials have such a low activation energy^36−38^ and does not seem to agree with the very low activation seen in these samples.

The fact that this material seems to be stable for a wide range of temperatures, is mechanically easier to prepare than most fluorite-based materials, and has low activation energies for fluorine conduction near room temperature makes it very interesting for applications such as fluorine ion batteries. It is interesting to focus further in future studies on the ionic conduction mechanism to test if it could be decoupled from the electronic conductivity to allow for a wider range of applications.

## Conclusions

SrF_2_–TiO_2_ composite materials have exhibited very interesting reactions, showing that some compositions form TiO_*x*_F_*y*_ and also that there are changes in the behavior of the SrF_2_ component. Materials based on this combination have shown very high conductivities for fluorine conduction due to fluorine moving from the SrF_2_ component to the TiO_2−δ_ phase. Up to 25% TiO_2_ can be added with little to no TiO_2_ phase remaining, while maximum conductivity is observed with 33% TiO_2_. Further increasing the TiO_2_ content increases the electronic conductivity without increasing the ionic conductivity. Such materials can be interesting if control over the electronic/ionic conductivity ratio can be obtained by further studies.

## Supporting Information Available

EIS results for the analysis and a table with the value of each element in the circuit for every sample for every temperature (PDF)
