# Supplementary material for: Evidence of High Fluorine Ion Conductivity in SrF2-Rich SrF2–TiO2-Based Compounds
Source: ACS Omega. 2025 Apr 28;10(18):19214–23. doi: 10.1021/acsomega.5c02253 (PMC12079593; doi:10.1021/acsomega.5c02253)
Supplement: Supplementary file 1 — ao5c02253_si_001.pdf [file ao5c02253_si_001.pdf]

Evidence of high fluorine ion conductivity in SrF_2-rich SrF_2-TiO_2 based compounds.

Yatir Sadia*[a][b][c] Gwilherm Kerherve[c] and Stephen J. Skinner[c]

[a] Material Engineering, Ben Gurion University of the Negev, P.O.B. 653 Beer-Sheva 8410501 Israel
[b] Bar Ilan University, Faculty of Engineering Materials Engineering, Ramat Gan, 5290002
[c] Department of Materials Imperial College of London South Kensington Campus, London, SW7 2AZ, United Kingdom

*E-mail: yatir@bgu.ac.il, y.sadia@imperial.ac.uk

Supporting information:

Table SI1: The fitting parameters for the EIS analysis of the silver coted samples:

Sample	Temp:	L1 [H]	R1 [Ωcm]	R2 [Ωcm]	CPE -T [F]	CPE -P	C1- [F]
5 SrF_2: 1TiO_2	50°C	$3.3625x10^{-5}$	3530	2008	$4.255x10^{-9}$	0.82185	$6.309x10^{-11}$
	100°C	$2.0227x10^{-5}$	1682	1377	$3.278x10^{-9}$	0.84022	$7.976x10^{-11}$
	150°C	$1.3031x10^{-5}$	849.6	1067	$2.482x10^{-9}$	0.84926	$9.927x10^{-11}$
	200°C	$8.9012x10^{-6}$	445	1001	$2.170x10^{-9}$	0.83699	$1.226x10^{-10}$
	250°C	$5.2192x10^{-6}$	185.3	223.9	$4.340x10^{-15}$	0.46277	$2.44x10^{-10}$
4 SrF_2: 1TiO_2	50°C	$1.5725x10^{-5}$	947.1	1193	$1.1755x10^{-8}$	0.83517	$1.147x10^{-10}$
	100°C	$8.8698x10^{-6}$	409.8	735.9	$8.474x10^{-9}$	0.85301	$1.666x10^{-10}$
	150°C	$5.3234x10^{-6}$	181.7	544.6	$5.164x10^{-9}$	0.87115	$2.748x10^{-10}$
	200°C	$3.383x10^{-6}$	80.71	674.7	$1.996x10^{-9}$	0.88822	$5.009x10^{-10}$
3 SrF_2: 1TiO_2	50°C	$6.3132x10^{-5}$	277.6	467.2	$2.3192x10^{-8}$	0.81819	$2.421x10^{-10}$
	100°C	$4.1683x10^{-6}$	132	358.7	$1.9161x10^{-8}$	0.82106	$3.977x10^{-10}$
	150°C	$2.9212x10^{-6}$	67.46	378.8	$1.1908x10^{-8}$	0.82939	$6.684x10^{-10}$
2 SrF_2: 1TiO_2	50°C	$2.9195x10^{-5}$	99.72	114.6	$4.9717x10^{-8}$	0.84433	$6.245x10^{-10}$
	100°C	$1.97x10^{-6}$	43.23	81.19	$3.852x10^{-8}$	0.84969	$1.407x10^{-9}$
	150°C	$1.4998x10^{-6}$	19.78	103	$2.6665x10^{-8}$	0.84228	$3.253x10^{-9}$
1 SrF_2: 1TiO_2	50°C	$9.5436x10^{-7}$	8.297	12.44	$2.1973x10^{-7}$	0.8661	$2.2323x10^{-8}$
